# Serum contactin-1 as a biomarker of long-term disease progression in natalizumab-treated multiple sclerosis

**DOI:** 10.1177/13524585211010097

**Published:** 2021-04-23

**Authors:** Zoë YGJ van Lierop, Luuk Wieske, Marleen JA Koel-Simmelink, Madhurima Chatterjee, Iris Dekker, Cyra E Leurs, Eline AJ Willemse, Bastiaan Moraal, Frederik Barkhof, Filip Eftimov, Bernhard MJ Uitdehaag, Joep Killestein, Charlotte E Teunissen

**Affiliations:** Department of Neurology, Amsterdam UMC, Vrije Universiteit Amsterdam, MS Center Amsterdam, Amsterdam Neuroscience, Amsterdam, The Netherlands; Department of Neurology and Neurophysiology, Amsterdam UMC, Academisch Medisch Centrum, Amsterdam Neuroscience, Amsterdam, The Netherlands; Department of Clinical Chemistry, Amsterdam UMC, Vrije Universiteit Amsterdam, Neurochemistry Laboratory and Biobank, Amsterdam Neuroscience, Amsterdam, The Netherlands; Department of Clinical Chemistry, Amsterdam UMC, Vrije Universiteit Amsterdam, Neurochemistry Laboratory and Biobank, Amsterdam Neuroscience, Amsterdam, The Netherlands; Department of Neurology, Amsterdam UMC, Vrije Universiteit Amsterdam, MS Center Amsterdam, Amsterdam Neuroscience, Amsterdam, The Netherlands/Department of Rehabilitation Medicine, Amsterdam UMC, Vrije Universiteit Amsterdam, Amsterdam, the Netherlands; Department of Neurology, Amsterdam UMC, Vrije Universiteit Amsterdam, MS Center Amsterdam, Amsterdam Neuroscience, Amsterdam, The Netherlands; Department of Clinical Chemistry, Amsterdam UMC, Vrije Universiteit Amsterdam, Neurochemistry Laboratory and Biobank, Amsterdam Neuroscience, Amsterdam, The Netherlands; Department of Radiology and Nuclear Medicine, Amsterdam UMC, Vrije Universiteit Amsterdam, MS Center Amsterdam, Amsterdam Neuroscience, Amsterdam, The Netherlands; Department of Radiology and Nuclear Medicine, Amsterdam UMC, Vrije Universiteit Amsterdam, MS Center Amsterdam, Amsterdam Neuroscience, Amsterdam, The Netherlands/Institutes of Neurology and Healthcare Engineering, University College London, London, UK; Department of Neurology and Neurophysiology, Amsterdam UMC, Academisch Medisch Centrum, Amsterdam Neuroscience, Amsterdam, The Netherlands; Department of Neurology, Amsterdam UMC, Vrije Universiteit Amsterdam, MS Center Amsterdam, Amsterdam Neuroscience, Amsterdam, The Netherlands; Department of Neurology, Amsterdam UMC, Vrije Universiteit Amsterdam, MS Center Amsterdam, Amsterdam Neuroscience, Amsterdam, The Netherlands; Department of Clinical Chemistry, Amsterdam UMC, Vrije Universiteit Amsterdam, Neurochemistry Laboratory and Biobank, Amsterdam Neuroscience, Amsterdam, The Netherlands

**Keywords:** Multiple sclerosis, contactin 1, disease progression, disease activity, natalizumab, prediction

## Abstract

**Background:**

Natalizumab treatment provides a model for non-inflammation-induced disease progression in multiple sclerosis (MS).

**Objective:**

To study serum contactin-1 (sCNTN1) as a novel biomarker for disease progression in natalizumab-treated relapsing-remitting MS (RRMS) patients.

**Methods:**

Eighty-nine natalizumab-treated RRMS patients with minimum follow-up of 3 years were included. sCNTN1 was analyzed at baseline (before natalizumab initiation), 3, 12, 24 months (M) and last follow-up (median 5.2 years) and compared to 222 healthy controls (HC) and 15 primary progressive MS patients (PPMS). Results were compared between patients with progressive, stable, or improved disability according to EDSS-plus criteria.

**Results:**

Median sCNTN1 levels (ng/mL,) in RRMS (baseline: 10.7, 3M: 9.7, 12M: 10.4, 24M: 10.8; last follow-up: 9.7) were significantly lower compared to HC (12.5; *p* ⩽ 0.001). It was observed that 48% of patients showed progression during follow-up, 11% improved, and 40% remained stable. sCNTN1 levels were significantly lower in progressors both at baseline and at 12M compared to non-progressors. A 1 ng/mL decrease in baseline sCNTN1 was consistent with an odds ratio of 1.23 (95% confidence interval 1.04–1.45) (*p* = 0.017) for progression during follow-up.

**Conclusion:**

Lower baseline sCNTN1 concentrations were associated with long-term disability progression during natalizumab treatment, making it a possible blood-based prognostic biomarker for RRMS.

## Introduction

Multiple sclerosis (MS) is a chronic inflammatory disease of the central nervous system (CNS) leading to demyelination and neurodegeneration.^1^ It is the most common cause of chronic neurological disability in young adults in developed countries.^2^ Initially most patients have a relapsing-remitting disease course (RRMS), which converts to the secondary progressive stage after variable periods of time, characterized by gradual disability accumulation over a period of many years.^3^ The increased availability of effective disease-modifying therapies (DMTs) has led to the use of “no evidence of progression or active disease (NEPAD)” criteria to define treatment efficacy.^4^ Furthermore, sustained improvement in physical disability has been proposed as a desired treatment outcome.^5,6^ The interpretation of disability measures should take into account the expected inflammation reduction during the initial treatment phase, which potentially reverses disease worsening caused by relapses in the year prior to treatment initiation.^7^ The term “disease progression” is reserved for patients with ongoing disability advancement in spite of effective suppression of inflammatory disease activity by DMT.^8–10^

This phenomenon even occurs under the highly effective compound natalizumab (NTZ; Tysabri, Biogen Inc, Cambridge, MA, USA).^6,11,12^ When correcting for the expected reduction in inflammation, ongoing disease progression has been reported in a substantial portion of patients in the long term (64.6%, median follow-up (FU) of 4.9 years).^13^

There is an urgent need for objective and accessible biomarkers to use at an early stage for the monitoring or even prediction of disease progression. Such biomarkers should help to distinguish progressive disease from non-progressive disease independently from the coexistence of active inflammatory disease. Since (active) inflammation is suppressed during NTZ treatment, we propose that the disability development during NTZ treatment provides a model for non-inflammation-induced disease progression in MS. Using this model and the most studied blood-based biomarker in MS, serum neurofilament light (sNfL), this distinction could not be made.^14^

Contactin-1 (CNTN1) is a member of the contactin family, proteins that are important for the function and maintenance of myelinated neurons.^15,16^ CNTN1 is specifically expressed in paranodal axonal domains and involved in myelin formation in the CNS due to its role in axo–glia interaction, the loss of which forms an important cause of neuronal dysfunction and subsequent axonal loss in MS.^17–19^ Although primarily expressed in the CNS, CNTN1 is present in the peripheral nervous system (PNS) as well.^20,21^

We recently showed that MS patients had reduced CNTN1 concentrations in cerebrospinal fluid (CSF) compared to healthy controls (HC).^22^ Furthermore, CNTN1 concentrations in CSF were positively correlated with normalized brain volume and negatively correlated with T2 lesion load in secondary progressive MS (SPMS) patients, suggesting that CNTN1 is involved in disease progression.^22^ In a pilot study, we observed reductions in serum levels of CNTN1 (sCNTN1) in MS as well.^23^ We, therefore, hypothesize that sCNTN1 levels are changed due to underlying axonal dysfunction, and could be a novel blood-based biomarker to monitor disease progression. We here aim to study sCNTN1 as prognostic biomarker for disease progression in a long-term FU study in well-monitored NTZ-treated MS patients and investigate changes in sCNTN1 levels over time in relation to clinical and radiological progression.

## Methods

### Cohort

MS patients (*n* = 89) were selected from an ongoing prospective observational cohort study, which was initiated in 2006 at the VU University Medical Center Amsterdam. Patients were included if they were 18 years or older at initiation of NTZ and had a minimum FU of 3 years. Last FU was defined as the last infusion of NTZ before discontinuation of NTZ or clinical database closure for this project in November 2020. Clinical assessments were performed on a yearly basis and included relapse history, Expanded Disability Status Scale (EDSS) assessment by trained personnel, timed 25-foot walk test (T25W) and 9-hole peg test (9HPT). Blood samples were collected at baseline (BL) before the first NTZ infusion and every 3 months thereafter.

### Disability status definition

Patients were retrospectively divided into three “EDSS plus” categories based on worsening, stability, or improvement during FU of the EDSS, T25W, and/or 9HPT measurements.^24^ EDSS plus “progression” was defined as significant worsening on at least one of three outcome measurements. EDSS plus “improvement” was defined as significant improvement of one or more outcome measurement without worsening in other measurements. EDSS plus “stability” was defined as no significant changes in any measurement. Furthermore, patients with EDSS plus stability or improvement were referred to as “non-progressors” together. The threshold for significant EDSS progression (increase) or improvement (decrease) was at 1.5, 1, or 0.5 in case of a reference EDSS of 0, 1–5 or ⩾ 5.5, respectively. For T25W and 9HPT, the threshold was at 20% change. For 9HPT and T25W, we used the mean value of the dominant and non-dominant hand of two attempts.^24^ If the EDSS, T25W, or 9HPT changed above these thresholds, the change had to be confirmed during at least one following yearly visit before disability status was assigned. Cases with conflicting outcomes due to small fluctuations of EDSS scores or discrepant clinical measures were discussed in a clinical panel (J.K. and Z.Y.G.J.v.L.) blinded for sCNTN1 results to assign the final disability outcome.

### No evidence of disease activity and NEPAD status definition

To explore relations between CNTN1 levels and treatment response, we evaluated no evidence of disease activity (NEDA)-3 status (no relapses, no EDSS worsening, no evidence of radiological disease activity) at FU in every patient.^25^ We corrected for residual inflammation by excluding relapses that occurred in the first 3 months and by using brain magnetic resonance imaging (MRI) at year 1 as a reference. Relapses were defined as new neurological symptoms observed by a neurologist, lasting more than 24 hours and not attributable to other causes than MS. Radiological disease activity was defined as any new or enlarging (> 50% increase in size) T2 lesions or any new T1 gadolinium-enhanced (GE) lesions.

NEPAD status was defined by no relapses, no EDSS plus progression (no significant increase in EDSS, 9HPT, and T25W), and no evidence of radiological disease activity.

### Contactin-1 analysis

Blood was centrifuged within 2 hours (1800 g, 10 minutes at room temperature) and coded serum aliquots were stored at −80°C. sCNTN1 was measured from samples obtained at BL (before first NTZ infusion), 3 months (3M), 12 months (12M), and 24 months (24M) of NTZ treatment, and last FU. Analysis took place at the Neurochemistry lab of the Department of Clinical Chemistry (Amsterdam UMC location VUmc) on a Luminex^®^ platform according to the manufacturer’s instructions (Human Magnetic Luminex Assay, R&D systems, Minneapolis, USA). The sCNTN1 assay was analytically validated in-house prior to use according to standardized international protocols.^26^ Validation parameters are summarized in a supplementary file. Samples were randomized and analyzed in duplicates, blinded for disability status. Measurements with a coefficient of variation (CV) > 15% and outliers were repeated and were excluded from analyses if the CV remained > 15% (details in the “Results” section). Levels in MS patients were compared to a reference cross-sectional dataset of 222 HC described elsewhere.^21^ Furthermore, we compared sCNTN1 levels in RRMS and HC to a cohort of 15 primary progressive MS (PPMS) patients selected from the Amsterdam MS Biobank initiated in 2018. The first sample was taken prior to ocrelizumab initiation (BL), the first FU sample (FU1) was taken prior to the second 300 mg dose (2 weeks after BL), and the second FU sample (FU2) prior to the first 600 mg dose (6 months after BL).

### Brain MRI acquisition

MRI protocols were previously described^13^ and were acquired following the Magnetic Resonance Imaging in Multiple Sclerosis (MAGNIMS) expert panel guidelines.^27^ Briefly, this included both two-dimensional (2D) proton-density (PD)/T2-weighted and postcontrast T1-weighted images, both with a 3-mm slice thickness. Parameters of interest were radiological disease activity (new/enlarged T2 hyperintense lesions and/or T1 GE lesions) collected on a yearly basis after the initial BL scan (<3 months of NTZ initiation).

### Statistical analyses

Statistics were performed in IBM SPSS Version 26. BL characteristics (presented with *p*-value and a significance level of 0.05) were compared between the three disability groups (progressors, stable patients, and improvers) using Chi-square test for categorical variables (gender, occurrence of relapse(s), presence of radiological activity, NEDA-3, and NEPAD status). Kruskal–Wallis test with Bonferroni correction was used to compare continuous variables across groups, for these were not normally distributed (age, disease duration, number of relapses, MRI lesion numbers, EDSS, 9HPT, T25W, and sCNTN1 concentrations). Cross-sectional median sCNTN1 levels were compared between the MS subtypes and HC using Mann–Whitney *U* test. For comparison between the RRMS disability groups, Kruskal–Wallis test with Bonferroni correction was applied. Correlation analysis between sCNTN1 levels, age, disease duration, and EDSS were performed using Spearman’s correlation coefficient. In the RRMS cohort, median sCNTN1 levels at BL, 3M, 12M, and last FU were compared between patients with or without relapses during the corresponding period (1 year prior to BL, between BL and 3M, between 3M and 12M, and between 12M and last FU, respectively) using Kruskal–Wallis test. The same comparison was made between patients with or without radiological disease activity on MRI taken at BL, 12M, and between 12M and last FU, respectively. Repeated-measures Friedman test was applied to assess the variance in sCNTN1 between timepoints. Binary logistic regression analysis was applied to assess the predictive value (represented as odds ratio (OR) and 95% confidence interval (CI)) of BL sCNTN1 levels for disability progression (progressor versus non-progressor group, that is, stable and improver group combined). Prediction modeling was carried out by backward selection based on *p* < 0.10 on BL characteristics (gender, age, disease duration, number of relapses 1 year prior to BL and in the first year of FU, radiological disease activity at BL and during first year of FU, EDSS, 9HPT, T25W, and sCNTN1 levels at BL and 3M) and an area under the curve (AUC) was calculated.

### Ethical considerations

The local medical and biobank ethics committee approved this observational cohort study, and all subjects gave written informed consent for the collection and use of medical data and biological fluids for research purposes. This study adhered to the ethical principles of the Declaration of Helsinki.

## Results

### Clinical and radiological data

During the study period, progression, stability, and improvement in EDSS scores was found in 30%, 52%, and 18% of RRMS patients (*n* = 89), respectively. Based on EDSS plus definitions, 43 patients (48.3%) were assigned “progressors,” 36 (40.4%) “stable,” and 10 (11.2%) “improvers.” Improvers were significantly younger compared to stable patients and progressors, and also had a shorter disease duration at BL compared to stable patients (Table 1). BL clinical measures did not differ across groups, except for the 9HPT. Clinical and radiological parameters of disease activity showed no significant differences across the groups, either at BL or during FU.

**Table 1.** Clinical and radiological data for total cohort and subgroups: progressors versus stable versus improvers.

Clinical and radiological data	Total cohort (*n* = 89)	Progressor (*n* = 43)	Stable (*n* = 36)	Improver (*n* = 10)	*p*-value
Total cohort (%)	100	48	40	11	–
Females (%)	74	72	79	67	NS
Age (years)	38 (30–43)	40 (31–44)^a,b^	38 (33–43)^a^	27 (23–37)^a,b^	0.02^a^/0.007^b^
Disease duration (years)	7.4 (3.8–12.1)	7.4 (4.2–12.7)	8.9 (4.8–13.3)^a^	3.2 (1.0–7.5)^a^	0.016
Years of clinical FU	7.1 (4.9–10.3)	8.2 (5.6–11.4)	7.1 (4.3–9.9)	5.0 (4.4–8.8)	NS
Years from BL to last FU sample	5.2 (4.3–6.8)	5.7 (5.1–7.1)^a^	5.1 (4.1–6.5)	4.3 (3.4–5.7)^a^	0.024
With relapses (%)					
1 year prior to BL	85	86	82	89	NS
Year 1	16	21	11	10	NS
After year 1	10	9	6	30	NS
BL MRI					
With T1 GE (%)	68	66	71	67	NS
T1 GE number	2 (0–6)	1 (0–4)	3 (0–7)	1 (0–12)	NS
With T2 load > 38 (%)	65	64	71	44	NS
T2 load if < 38	26 (14–30)	29 (12–34)	26 (20–24)	16 (8–28)	NS
Radiological activity (%)					
During year 1	31	34	28	30	NS
After year 1	10	12	11	0	NS
BL disability					
EDSS	3.5 (2.5–5.0)	3.5 (2.5–5.5)	3.0 (2.5–5.0)	4.0 (3.3–5.3)	NS
9HPT (seconds)	22 (20–26)	22 (21–26)	20 (19–24)^a^	27 (21–42)^a^	0.023
T25W (seconds)	4.9 (3.9–7.2)	5.1 (4.3–8.2)	4.6 (3.5–5.6)^a^	5.9 (4.7–10.1)^a^	NS
Year 1					
EDSS	3.5 (2.5–4.5)	3.5 (3.0–5.5)	3.0 (2.5–4.0)	3.75 (3.0–4.4)	NS
9HPT (seconds)	22 (20–26)	23 (20–26)	20 (19–25)	24 (20–34)	NS
T25W (seconds)	4.8 (3.9–6.1)	5.1 (4.2–7.7)	4.6 (3.4–5.8)	4.6 (3.7–7.8)	NS
Last FU					
EDSS	4.0 (3.0–5.5)	5.0 (3.5–6.0)^a,b^	3.5 (2.0–4.0)^a^	2.5 (1.5–3.9)^b^	0.001^a,b^
9HPT (seconds)	23 (20–28)	24 (20–31)^a^	20 (18–25)^a^	25 (20–40)	0.018
T25W (seconds)	5.0 (4.1–7.4)	5.6 (4.7–10.5)^a^	4.1 (3.5–5.5)^a^	5.3 (4.3–7.6)	0.000
NEDA-3 during FU (%)	47	21	72	70	0.000
NEPAD during FU (%)	27	0	53	50	0.000

NS: not significant; FU: follow-up; BL: base line; MRI: magnetic resonance imaging; T1 GE: T1 gadolinium-enhanced lesion(s); T2: T2 hyperintense lesion; EDSS: Expanded Disability Status Scale; 9HPT: 9-hole peg test; T25W: timed 25-foot walk test; NEDA: no evidence of disease activity; NEPAD: no evidence of progression or active disease.

a The *p*-values are indicated for comparison between three groups.

b The *p*-values are indicated for comparison between two groups.

Mean values are presented with ±standard deviation, median values with interquartile ranges (IQR).

The PPMS cohort included 4 (27%) female patients. At the time of the first blood sample for sCNTN1 analysis, median age was 52 years (50–60), median disease duration 7.3 years (3.2–13.1), and median EDSS 5.5 (4–6.5).

### Serum CNTN1

**Figure 1.**
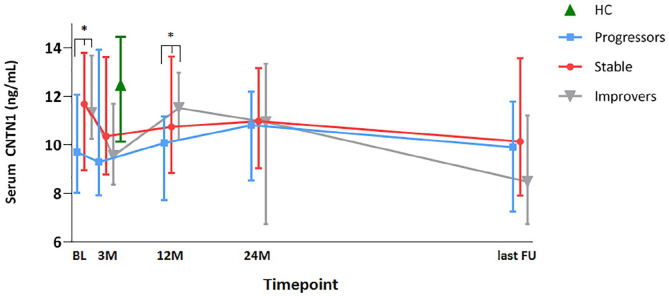
Serum CNTN1 levels at each timepoint (ng/mL; symbols are placed at the median values and vertical bars indicate interquartile range (IQR)) separated by disability group. Disability categories were defined by EDSS plus (EDSS combined with 9HPT and T25W) criteria as follows: patients with progressive disability (“progressors,” *n* = 43), patients with “stable” disability (*n* = 36), or improved (“improvers,” *n* = 10) disability. Samples were taken at baseline (BL, before natalizumab initiation), after 3 months (3M), 12 months (12M), 24 months (24M), and at last follow-up (last FU). Healthy control (HC) levels (only measured once) are illustrated in between these timepoints for the purpose of clarity of this graph. The asterisks (*) indicate significant differences in sCNTN1 levels found between progressors and non-progressors (stable patients and improvers together).

Assay validation parameters are presented as supplemental material. sCNTN1 was analyzed in a total of 442 RRMS patient samples. Three patients had one missing sample during FU. Results from five additional samples were excluded because of intra-assay CV for the sCNTN1 result > 15%, leading to inclusion of 89 (BL), 87 (3M), 83 (12M), 85 (24M), and 88 (last FU) sCNTN1 results in the analyses. Median sCNTN1 (IQR) concentrations (ng/mL) at each timepoint in the total RRMS cohort and for each disability group are presented in Figure 1.

**Figure 2.**
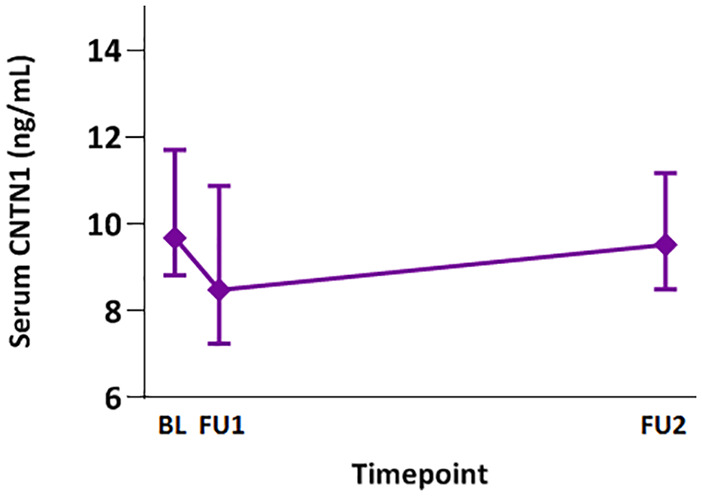
Serum CNTN1 levels (ng/mL; symbols are placed at the median values and vertical bars indicate interquartile range (IQR)) in primary progressive MS measured prior to ocrelizumab initiation (BL), prior to the second 300 mg dose (FU1) and prior to the first 600 mg dose (FU2). All levels were significantly lower compared to HC (*p* = 0.002) and a significant decrease was found between BL and FU1 timepoint (*p* = 0.011).

In the PPMS cohort (*n* = 15), sCNTN1 was analyzed in 43 samples. Median sCNTN1 levels at the three timepoints are presented in Figure 2.

### Cross-sectional sCNTN1 analyses

#### MS patients versus HC

Median sCNTN1 concentrations were significantly lower in MS compared to HC (12.47 ng/mL (IQR: 10.14–14.46)), in the total MS cohort (*p* = 0.000) and in progressors (*p* ⩽ 0.000) specifically (Figure 1). sCNTN1 levels in stable MS patients were similar to HC at BL, but were significantly lower at 3M (*p* = 0.008), 12M (*p* = 0.039), 24M (*p* = 0.035), and last FU (*p* = 0.003). In the improver group, sCNTN1 levels at 3M (*p* = 0.006) and last FU (*p* = 0.001) were significantly lower compared to HC. The other timepoints in the improver group did not differ from those in HC. In the PPMS cohort, sCNTN1 levels were significantly lower compared to HC (*p* = 0.002), before and during B-cell depletion due to treatment with ocrelizumab.

#### Relations with clinical and radiological data

No significant correlations were found between BL sCNTN1 levels and age or disease duration at BL in the RRMS patients. The sCNTN1 levels at BL, 3M, 12M, 24M, and last FU did not significantly differ between RRMS patients with or without relapses or radiological disease activity during a corresponding period (1 year prior to BL, BL–3M, 3–12M, 12–24M, and 24M to last FU, respectively). The sCNTN1 levels were neither different between patients who remained NEDA-3 and/or NEPAD and patients who lost NEDA-3 and/or NEPAD status during FU.

Within the PPMS cohort, no correlations were found between sCNTN1 levels and sex, age, disease duration, and EDSS, respectively.

#### Differences across disability groups

Median sCNTN1 levels were significantly lower in progressors both at BL (9.71 ng/mL, interquartile range (IQR: 8.03–12.07), *p* = 0.017) and at 12M (10.08 ng/mL, (IQR: 7.7–11.18), *p* = 0.045) compared to non-progressors, that is, stable patients and improvers together (BL: 11.61 ng/mL, (IQR: 9.85–12.69); 12M: 10.99 ng/mL, (IQR: 8.97–12.64)). No significant differences were found at the other timepoints (3M, 24M, and last FU) between the two groups, nor at any timepoint across three disability groups (progressors versus stable patients versus improvers).

The PPMS cohort had a significantly higher age and BL EDSS compared to the RRMS cohort. BL and FU1 sCNTN1 levels in the PPMS cohort were significantly lower compared to RRMS non-progressors at BL (*p* = 0.048) and 3M (*p* = 0.046), but this difference lost significance after correcting for age.

### Longitudinal sCNTN1 analyses

#### Differences between timepoints

The sCNTN1 levels did not vary over time. More in detail, no significant differences were found in sCNTN1 between subsequent timepoints in the total cohort or within disability groups. In the total RRMS cohort, no significant difference was found between BL and 3M sCNTN1 level. Within disability groups, only improvers showed a significant decrease (*p* = 0.037). Similarly, within the PPMS patients, a decrease was observed between BL and FU1 sCNTN1 levels (*p* = 0.011; Figure 2).

#### Prediction of disease progression in RRMS pati-ents

Lower sCNTN1 levels at BL were associated with disability progression during FU in RRMS patients under NTZ (OR: 1.23; 95% CI: 1.04–1.45; *p* = 0.017; for every one-point (ng/mL) decrease in BL sCNTN1). This association withstood correction for age at start NTZ and number of relapses during first year of FU (OR: 1.28, 95% CI: 1.066–1.540, *p* = 0.008). The best possible prediction model for disability progression in this cohort included BL sCNTN1 (β = −0.248, *p* = 0.008), age at start NTZ (*p* = 0.089), and relapses during first year of FU (*p* = 0.072), with an AUC of 0.693.

## Discussion

In this study, we used NTZ treatment as a model to investigate serum CNTN1 as a biomarker for monitoring and prediction of disease progression independent of inflammation. We found that sCNTN1 levels were lower in both RRMS and PPMS patients compared to HC. Next, we found that RRMS patients with disability progression during FU (progressor group) had a significantly lower sCNTN1 level at BL (before NTZ initiation) and after 12 months of treatment compared to non-progressors. Until 24 months of NTZ treatment, sCNTN1 levels remained relatively lower in progressors compared to the other groups, even though the difference at 24 months was not significant in a direct comparison. Moreover, BL sCNTN1 was a predictor of future disease progression, independent of the number of relapses during first year of FU and age at BL. sCNTN1 levels in this study did not vary significantly between timepoints in the total cohort or within a specific disability group. Between BL and 3M timepoint, we found a significant decrease in sCNTN1 in improvers only. A similar trajectory was found in PPMS patients. Even though the cohort was small, this tentatively confirms the relation between CNTN1 and disease progression and the response of this marker to highly effective DMT.

To our knowledge, there are no previous studies on the longitudinal development of sCNTN1 in MS to compare our results with. The stable CNTN1 levels in serum are seemingly in contrast to the trend toward higher cross-sectional levels in CSF of SPMS and PPMS patients compared to RRMS that has been reported earlier.^22^ However, the lower cross-sectional sCNTN1 levels in our RRMS cohort compared to our HC cohort are in accordance with several previous studies. For example, CSF proteomics studies^28,29^ have reported significantly decreased CSF CNTN1 levels in RRMS patients compared to clinically isolated syndrome (CIS) patients and controls and in neuromyelitis optica patients compared to controls.^30^ In addition, studies focusing on CNTN1 analysis in CSF^22,23^ and serum^22,23^ of MS patients and chronic inflammatory demyelinating polyneuropathy also observed decreased levels.^21^ Just one other CSF proteomics study has reported contrasting results, with increased levels in secondary progressive MS (SPMS) patients compared to control subjects and in CIS patients compared to other neurological disease.^31^ The exact relation of CNTN1 in blood versus CSF levels and their specificities for each disease stage in MS remains unclear and should be further studied.

The exact processes behind the changes over time in CNTN1 are not yet known. Axonal damage that underlies disease progression in MS could cause increased release of CNTN1 in body fluids during the active phase, whereas decreased CNTN1 levels could reflect the reduced axonal density that is found in progressive MS patients^32,33^ or reflect reduced CNTN1 RNA transcription. However, in vitro and in vivo models of MS have linked an increased axonal expression of CNTN1 to the signaling pathways involved in remyelination.^34,35^ Consequently, lower levels of CNTN1 in CSF and serum could be a consequence of active retention of CNTN1 into axons to support myelination and axonal repair and, therefore, reflect a restorative mechanism. Following this theory, the lower sCNTN1 levels we found for all timepoints compared to HC, as well as the decrease between BL and 3M timepoints in improvers and between BL and FU1 timepoints in PPMS, could reflect a compensatory mechanism for axonal damage that occurred pre-BL. Furthermore, the fading of this difference among disability groups under highly effective DMT could reflect successful compensatory mechanisms.

The high proportion (48%) of EDSS plus progression (EDSS, 9HPT, and T25W combined) found in the NTZ-treated RRMS patients during this study is consistent with previous reports on RRMS patients with effective DMT.^8,9,13^ We further categorized non-progressors into stable patients and improvers and found improvers were significantly younger with shorter disease duration at BL, which is in line with previous findings and attributed to relatively less irreversible damage but more pronounced disease activity.^36–38^ BL inflammatory disease activity was equally present across disability groups and showed comparable, low rates of residual inflammation during FU. This supports the emerging theory that silent progression occurs, at least in part, independent from inflammatory mechanisms and could be driven by more diffuse, possibly neurodegenerative injury.^8,39^ The lack of association between sCNTN1 levels and NEDA-3 or NEPAD status during FU do support that the difference in BL and 12M sCNTN1 levels is mainly driven by inflammation-independent mechanisms. Overall, our results show sCNTN1 could be a potential novel blood-based biomarker for various phases of disease progression in MS, although interpretation remains challenging at this point and further research is needed to unravel the mechanisms behind varying levels over time.

This study is not without limitations. We were restricted to number of focal brain MRI lesions because different scanners and protocols were applied during FU. Future investigation should, therefore, include longitudinal whole brain, regional and lesion volumes to shed more light on the role of sCNTN1 on various aspects of disease progression in MS. Furthermore, the relatively small group sizes could have impeded significance in our analyses. Studies in larger cohorts preferably on different DMT are needed to assess the added value of CNTN1 in monitoring and predicting disease progression in MS.

In conclusion, our study provided insight into longitudinal sCNTN1 levels in MS patients and showed that lower BL sCNTN1 concentrations were associated with long-term disability progression during NTZ treatment, making it a possible prognostic blood-based biomarker for disease progression in MS.

## Supplemental Material

sj-pdf-1-msj-10.1177_13524585211010097 – Supplemental material for Serum contactin-1 as a biomarker of long-term disease progression in natalizumab-treated multiple sclerosis Click here for additional data file. Supplemental material, sj-pdf-1-msj-10.1177_13524585211010097 for Serum contactin-1 as a biomarker of long-term disease progression in natalizumab-treated multiple sclerosis by Zoë YGJ van Lierop, Luuk Wieske, Marleen JA Koel-Simmelink, Madhurima Chatterjee, Iris Dekker, Cyra E Leurs, Eline AJ Willemse, Bastiaan Moraal, Frederik Barkhof, Filip Eftimov, Bernhard MJ Uitdehaag, Joep Killestein and Charlotte E Teunissen in Multiple Sclerosis Journal
