# Supplementary material for: Serum contactin-1 as a biomarker of long-term disease progression in natalizumab-treated multiple sclerosis
Source: Mult Scler. 2021 Apr 23;28(1):102–10. doi: 10.1177/13524585211010097 (PMC8689420; doi:10.1177/13524585211010097)
Supplement: sj-pdf-1-msj-10.1177_13524585211010097 – Supplemental material for Serum contactin-1 as a biomarker of long-term disease progression in natalizumab-treated multiple sclerosis [file sj-pdf-1-msj-10.1177_13524585211010097.pdf]

ASSAY VALIDATION REPORT

This report discusses the methods and results of the sCNTN1 assay validation of the Luminex® platform. Validation parameters are summarized in Table 2, detailed information and illustrations are presented below.

Validation parameter	Result	Acceptable range
LLOQ (pg/mL)	19.7	-
Intra-assay %CV	3.2	<15
Inter-assay %CV	13.9	<15
Linearity (%)	90.8	85-115
Parallelism (%)	167	85-115
Recovery (%)	77	85-115

Table 2. Analytical validation parameters of the Luminex® platform for serum CNTN1 analysis.

1. Sensitivity: the lowest amount of an analyte in a sample that can be quantitatively determined with suitable precision and accuracy.

For the determination of the lower limit of quantitation (LLOQ), 16 blank (assay diluent) samples were measured in one plate. The calibration curve was calculated using a four parameter logistic curve fit, which gave the most optimal fit. LLOQ: assay replicates of diluent, calculate LLOQ as 10 SD above the blank, resulting in a LLOQ of 19.7 pg/mL.

2. Precision: The variation in CNTN1 measurement within a single assay (repeatability, intra-assay variation) and between independent assays performed on different days (intermediate precision, inter-assay variation).

Intra-assay variation (repeatability) was determined by analysis of samples (n=38) in two replicates within one plate. The mean coefficient of variation (%CV) was calculated by averaging the CVs of all tested samples. Average intra-assay variation of duplicate measurements was well below the accepted cutoff of 15% CV (3.2% CV).

Inter-assay variation (intermediate precision) was measured to determine the variation of analyses between different days. To quantify inter-assay variation, samples with low, medium, and high concentrations were selected from the samples used for the intra-assay variation [quality control (QC) low, QC medium and QC high]. These samples were measured in duplicate over 40 different plates at identical positions in the assay plates on 20 different days. The mean %CV was calculated for all samples. Average inter-assay variation over all different days was well below the accepted cutoff of 15% CV (13.9% CV).

3. **Dilutional linearity: ability of the assay to accurately and reliably detect the CNTN1 in serum spiked with the calibrator at a very high concentration after dilution (hook effect) and the ability of endogenous CNTN1 in serum to be detected at various dilutions accurately and reliably.**

For this, three different serum samples were spiked with CNTN1 concentrations above the upper limit of quantification and serial diluted.
No hook effect, i.e., suppression of signal at concentrations above the upper limit of quantification, was observed. The assay showed an acceptable dilutional linearity of 90.8% over the longest dilution range (Figure 3).

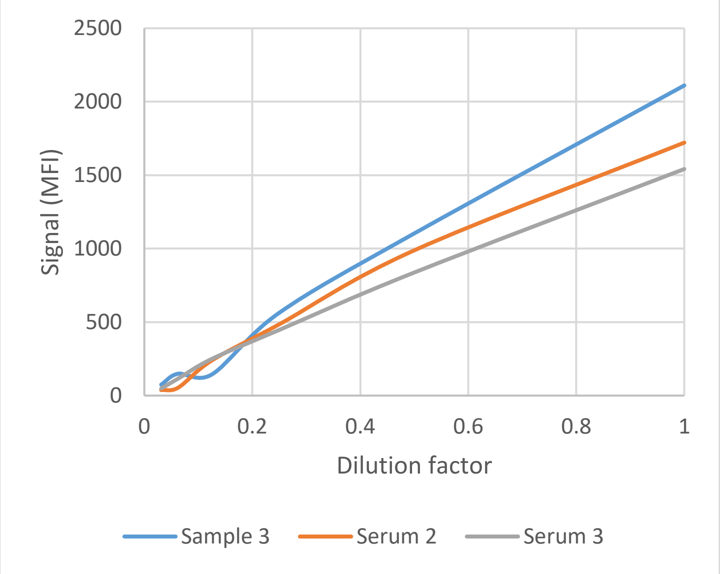

Figure 3. Linearity of the sCNTN1 assay.

4. Recovery: Ability to accurately and reliably measure the concentration of serum spiked with calibrator.

Four different serum samples were spiked with recombinant CNTN1 protein at concentrations across the range of the calibrator curve and serially diluted. The average %Recovery for these three samples was 77%.

5. Parallelism: Comparison of the signal vs. dilution factor response of the calibrator and endogenous CNTN1 in serum.

Four different serum samples, with high endogenous protein concentrations, were serially diluted. Both reciprocal relative dilution factor and MFI (Mean Fluorescent Intensity) signals of the samples and calibrator were log-transformed to be able to use linear regression to calculate the slopes of the sample and calibrator curves. The slope of the linear parts of the log–log transformed calibrator and sample dilution series were compared to determine the degree of parallelism by calculating the "in range%".

Parallelism between endogenous CNTN1 in serum samples and recombinant CNTN1 calibrator was not demonstrated by plotting the MFI signal against the dilution factor (reciprocal). The mean percentage parallelism of 167% was not within the predefined ranges of 85-115%, however samples were behaving parallel to each other.

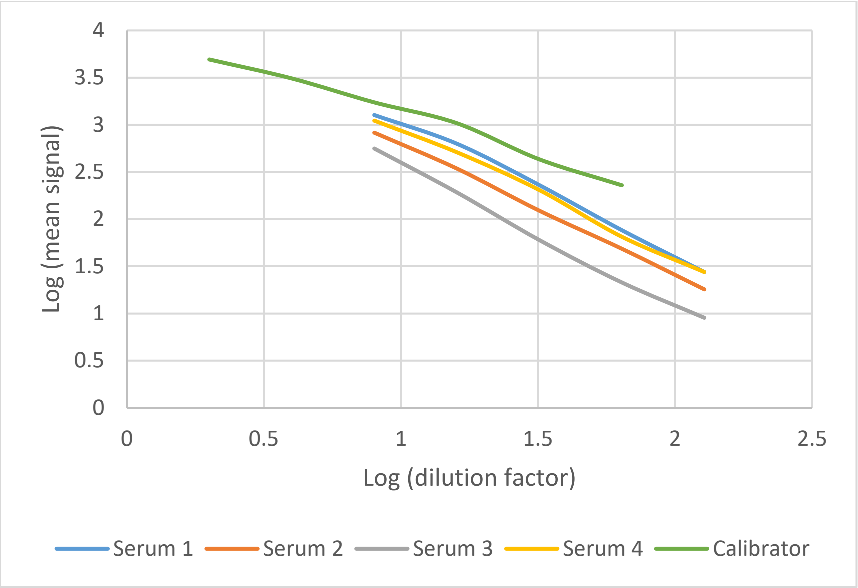

Figure 4. Parallelism of the sCNTN1 assay.

6. Freeze-thaw stability

The impact of repeated freeze–thaw cycles after processing was determined in 3 individual serum samples (Figure 5). The effect of repeated freeze–thaw cycles was studied by freezing the samples directly after centrifugation and thawing the samples up to 4 times extra by keeping them for 2 hours at room temperature and freezing it again at − 80 °C for a minimum of 24 hours. The reference sample was stored directly at − 80 °C. The CNTN1 levels were not affected significantly by freeze-thawing up to 4 cycles. Percent recovery compared to the reference sample was between 96 and 109% after up to 4 freeze-thaw cycles.

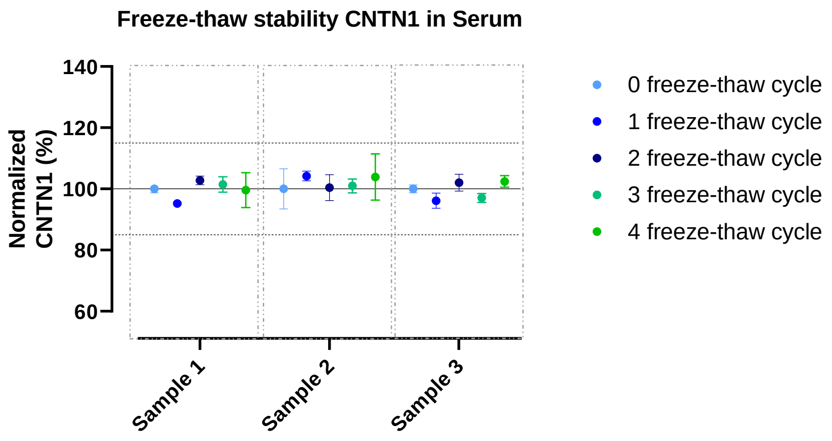

Figure 5. Impact of freeze-thaw cycles on sCNTN1 concentrations (pg/mL).
